# RPmirDIP: Reciprocal Perspective improves miRNA targeting prediction

**DOI:** 10.1038/s41598-020-68251-4

**Published:** 2020-07-16

**Authors:** Daniel G. Kyrollos, Bradley Reid, Kevin Dick, James R. Green

**Affiliations:** 1 Department of Systems and Computer Engineering, Carleton University, Ottawa, Canada; 2 Institute of Data Science, Carleton University, Ottawa, Canada

**Keywords:** Genomics, Transcriptomics

## Abstract

MicroRNAs (miRNAs) are short, non-coding RNAs that interact with messenger RNA (mRNA) to accomplish critical cellular activities such as the regulation of gene expression. Several machine learning methods have been developed to improve classification accuracy and reduce validation costs by predicting which miRNA will target which gene. Application of these predictors to large numbers of unique miRNA–gene pairs has resulted in datasets comprising tens of millions of scored interactions; the largest among these is mirDIP. We here demonstrate that miRNA target prediction can be significantly improved ($p < 0.001$) through the application of the Reciprocal Perspective (RP) method, a cascaded, semi-supervised machine learning method originally developed for protein-protein interaction prediction. The RP method, aptly named RPmirDIP, augments the original mirDIP prediction scores by leveraging local thresholds from the two complimentary views available to each miRNA–gene pair, rather than apply a traditional global decision threshold. Application of this novel RPmirDIP predictor promises to help identify new, unexpected miRNA–gene interactions. A dataset of RPmirDIP-scored interactions are made available to the scientific community at cu-bic.ca/RPmirDIP and 10.5683/SP2/LD8JKJ.

## Introduction

MicroRNAs (miRNAs) represent a class of short (18–28 nucleotide [nt]) non-coding RNA molecules. They achieve post-transcriptional and translational regulation of protein expression via base-pairing with complementary sequences of messenger RNA (mRNA) molecules.

Gene regulation by miRNAs does not adhere to a simple one miRNA–one target gene mapping. Rather, the distribution of predicted targets reflect commensurately more complicated miRNA–mRNA combinatorics: miRNAs exhibit *target multiplicity* wherein more than one mRNA is targeted per miRNA, and mRNAs exhibit *signal integration* wherein more than one miRNA may target an mRNA. Consequently, the multi-valency of miRNAs enable their targeting of multiple genes, thus regulating the expression of several proteins. These miRNAs play key roles in gene regulation and their dysregulation is associated with several diseases^1,2^. Studies have revealed miRNAs involved in disease pathogenesis^3^, biological development^4^, stress response^5^, and cell cycle control^6^. The elucidation of miRNAs within genomes is, thus, critical to understanding the underlying mechanisms of organismal biology and cellular function.

While wet-laboratory experimentation are traditionally used to identify miRNA target interactions and gene regulation, these methods are resource-intensive as compared to complimentary computational approaches. Common examples of wet-laboratory experiments used for miRNA target interactions include low-throughput methods such as quantitative polymerase chain reaction (qPCR), western blotting, and reporter gene assays; numerous computational approaches leverage the higher throughput methods which are based on cross-linking and immunoprecipitation (CLIP). Here, we provide a brief overview of contemporary wet-laboratory miRNA–mRNA interaction detection methods.

The qPCR and western blot methodologies are used to determine the change in mRNA or protein concentrations and are, therefore, considered indirect methods for the detection of physical interactions^7^. Reporter gene assays, such as the luciferase reporter assay, transfect miRNAs into a cell line that stably expresses a luciferase reporter containing the 3‘-UTR of the target miRNA being investigated in order to quantify the degree of interaction based on the change in reporter gene expression^8^. The reporter gene methods are generally more informative to elucidate specific miRNA–mRNA binding and are, therefore, considered a high-confidence measure of interaction^9^. The high-throughput sequencing of RNA isolated by CLIP (HITS-CLIP) is also used to investigate miRNA–mRNA interactions by probing for Argonaute-miRNA and Argonaute-mRNA interactions and overlaying the results to identify putative interactions^10^. Similar to the HITS-CLIP and CLIP-Seq methods is the photoactivatable-ribonucleoside-enhanced CLIP (PAR-CLIP) procedure which leverages a more efficient cross-linking to stabilize the protein-RNA complexes and identified RNA-binding proteins sites on the target RNAs^11^. Finally, the cross-linking ligation and sequencing of hybrids (CLASH) method, as its name indicates, is an experimental procedure to identify miRNA–mRNA interaction sites using cross-linking, ligation, and sequencing of hybrids^12^. Taken together, the low- and high-throughput wet-laboratory techniques provide an ensemble of methods to identify interacting miRNA–mRNA pairs amenable to developing learning algorithms capable of further exploring the space of possible pairs to identify putative interactions.

### Current state of miRNA–target prediction

Within humans alone, the number of known miRNA exceeds 2,300^13^ and the human mRNA population is estimated to exceed 25,000^14^; current wet laboratory technology is unable to feasibly test each possible miRNA–gene pair. Consequently, there is significant interest in using computational approaches for miRNA target prediction. These predictors are used to narrow the scope of potentially interesting interactions, functioning as a guide to wet-laboratory validation experiments to more rapidly elucidate gene regulation networks.

Many ancestral and popularized computational methods follow an *ab initio* approach, including miRanda^15^, TargetScan^16^, and PITA^17^. These methods compare the nucleotide sequences of the miRNA against a $\sim 20-30$nt region of the mRNA in a search for matching sub-sequences. These methods also incorporate other string-matching rules based on the observed deviations from Watson-Crick pairing rules, such as G:U wobble pairing^18^, originally discovered in early miRNA–mRNA experimental validations. miRanda uses a scoring matrix based on the complementarity of each nucleotide pair, where a set of weighted heuristic rules for each nucleotide pairing contribute to a summative score for a given interaction^15^. PITA leverages both structure- and sequenced-based information by computing fusing target-site accessibility (the energy required to access target binding sites within mRNA secondary structures) with sequence-based matching to determine an improved overall score^17^. TargetScan leverages a series of stepwise linear regression models to identify the most informative features from 74 datasets culminating into what the authors denote the context++ model, demonstrated to outperform preceding methods as of 2015^16^.

In the time since, increasingly accurate miRNA target prediction algorithms have emerged, many leveraging machine learning as reviewed in^19^. Notable examples of classical machine learning include TarPMir^20^, RFMirTarget^21^, and MirTarget^22^ with recent models leveraging deep learning, including MiRTDL^23^, DeepMirTar^24^, and miRAW^25^. MirTarget is a Support Vector Machine (SVM) trained on CLIP experimentally validated interactions and miRNA overexpression data. The miRNA overexpression data provides a complimentary view to understanding functional targets as the elucidation of target interaction does not necessarily result in gene down-regulation^22^. The miRTDL method implemented a Convolutional Neural Network (CNN) with selected features obtained from the convolved feature maps^23^. DeepMirTar used Stacked denoising Autoencoders (SdA) to learn a lower-dimensional representation of latent features^24^ while miRAW leveraged autoencoders without the denoising step^25^. TarPMir used a Random Forest (RF) classifier trained on an experimentally validated dataset^20^. RFMirTarget also used a Random Forest classifier, however it was trained on data originally pre-computed by miRanda, thus acting as a cascaded refinement of *ab initio* predictions^21^. While all methods reviewed here relate to miRNA target prediction, a subset formulate the problem for the identification of the binding site as distinctly different from scoring the likelihood of interaction between a given miRNA and target; this work focuses on the latter problem.

**Figure 1:**
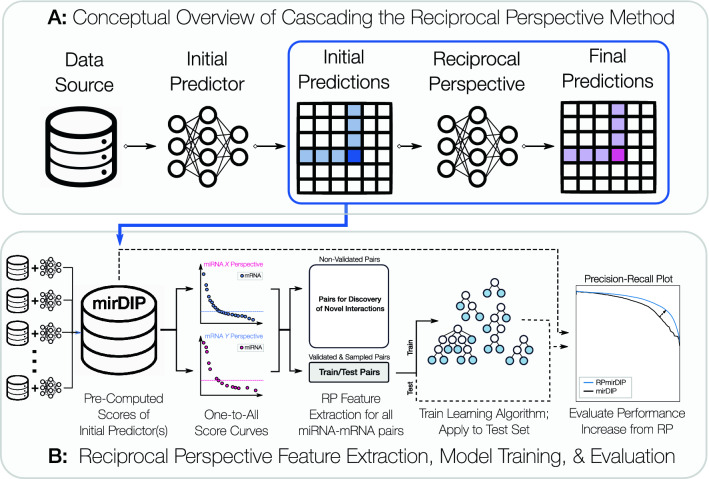
Conceptual overview of the Reciprocal Perspective algorithm applied to miRNA Target Prediction. Panel (**A**) depicts a high-level overview of cascading the Reciprocal Perspective learning algorithm based on an initial set of predictions from an initial learning algorithm. Panel (**B**) depicts an in-depth illustration of the combination of numerous individual miRNA targeting predictors to produce an ensembled mirDIP score. These scores are used to generate One-to-All score curves from which the Reciprocal Perspective features are extracted to train the cascaded learning algorithm and produce the final predictions for evaluation.

Pre-computed prediction databases facilitate access to predictions without having to execute predictive models. Conveniently, the predictions from multiple predictors have been aggregated in databases to generate a quantitative measure of confidence in a given miRNA–gene pair. The largest of such databases, both in number of integrated sources and total number of pairs, is the mirDIP pre-computed miRNA–target interaction database^9^. This dataset is a boon, not only to wet-laboratory experimentalists, but also to researchers seeking to develop new methods to further improve miRNA target prediction. Figure 1 provides a conceptual overview of how the mirDIP dataset was used to develop one such cascaded machine learning method.

### Reciprocal Perspective cascaded learning algorithm

**Figure 2:**
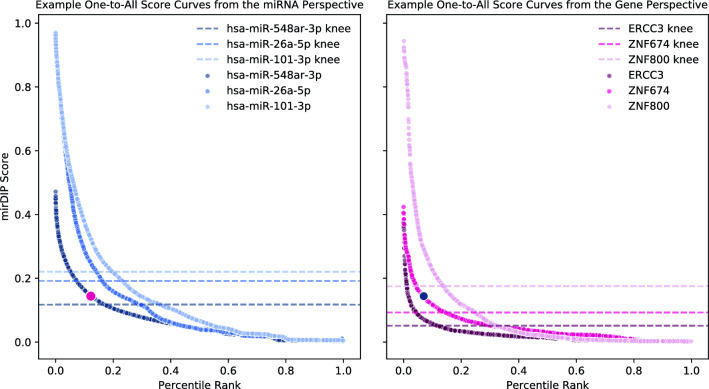
Example one-to-all score curves. In both perspectives, each point represents the mirDIP predicted score for one gene-miRNA pair. In the miRNA perspective (left), each curve represents all miRNA–gene pairs involving a specific miRNA (distinguished by shade), sorted by prediction score. The complimentary perspective (right) contains the rank order distribution of pairs involving individual genes (again distinguished by shade), where each data point is a specific miRNA. For each curve, the point identified as the knee is highlighted with a horizontal dashed line. The pair involving the hsa-miR-548ar-3p miRNA and ZNF674 gene is highlighted in each perspective with a larger marker and the corresponding colour. Since it falls above the knee in both perspectives, it will likely be scored highly by RPmirDIP.

The Reciprocal Perspective (RP) method was originally introduced as a cascaded, semi-supervised learning algorithm to improve the pair-wise predictive performance of existing learning algorithms within the context of protein-protein interaction (PPI) prediction^26^. Figure 1A provides a conceptual overview of how the scores produced by an initial predictor can be used to train a cascaded learning algorithm to provide refined prediction. Leveraging the output scores generated by an initial learning algorithm as input to the RP method, those scores are cast into a new rank-order domain denoted a One-to-All score curve (O2A; Fig. 2) which, in the case of miRNA targeting prediction, provides two complimentary views, an miRNA-based “perspective” and a gene-based “perspective” (Fig. 1B).

For a given query pair (*X*, *Y*), RP examines the pair’s predicted score in the context of all predicted scores for all pairs involving either $(X,*)$ or $(*,Y)$. By leveraging predictions made on pairs not definitively known to be positive or negative (i.e. unlabelled pairs), this method can be described as semi-supervised machine learning. Both labeled and unlabeled scored pairs are available for feature extraction and use as part of a cascaded learning algorithm. For example, by examining the O2A curve for a given miRNA, *X*, it is relatively straightforward to determine a suitable local decision threshold for all putative targets of that miRNA. By repeating this analysis for the given gene, *Y*, a local threshold can be determined for all miRNA that may interact with *Y*. In combination with the local threshold, these O2As enable the extraction of several additional context-based features from and between these two views for each (*X*, *Y*) to train the RP cascaded learning algorithm and refine the original predictions. Applied to two state-of-the-art PPI predictions over five organisms, the RP-augmented models produced a statistically significant improvement over all conditions^26^.

Cascaded prediction refinement techniques in miRNA–target prediction is common. For example, the RFMirTarget method^21^, previously discussed, improves the predictions produced by miRanda^15^ using an additional 34 sequence-based features in an RF model to refine predictions. The RP method differentiates itself from other cascaded predictors in that it is domain-agnostic; it derives features from a domain entirely independent of the context of the original problem, functioning as an error-correction process. This flexibility enables it to be applied broadly to pair-wise prediction tasks in combination with a given initial predictor.

However, in its original implementation, the RP algorithm required an initial predictor capable of generating a complete-graph of prediction scores; that is, generate a score for every possible pair producing a comprehensive prediction matrix (CPM). This is considered computationally intractable for the majority of predictors (typically too slow) or for the majority of tasks (typically too large). In this work, we not only seek to improve the state-of-the-art in miRNA prediction, but additionally demonstrate the applicability of RP to predictions tasks where the assumption of a complete CPM is relaxed. To the best of our knowledge, this work presents the first instance where the predicted outputs of miRNA–gene pairs in a (near) all-to-all context has been reported.

## Methods

The following section describes the acquisition of pre-computed miRNA–target prediction data (i.e. initial scores); the collection of experimentally validated miRNA–target interactions to be used for training and testing (ground truth labels that were experimentally validated only after the generation of initial scores); the adaptations to the RP learning algorithm for this task; and the implementations of the RP cascaded model. Figure 1 depicts the conceptual overview of the prediction pipeline including data acquisition, RP feature extraction, training of a cascaded learning algorithm, and performance evaluation. Briefly, the prototypical prediction pipeline will use a data source to train and evaluate a predictor and generate a set predictions. The RP method leverages these predicted scores as a data source (i.e. in a cascade) to train and evaluate the RP model to generate a final set of predictions (Fig. 1A).

### Acquisition of the miRNA–target prediction data

The miRNA–target prediction data was acquired from the mirDIP database. This database contains 41 million unique miRNA–gene interactions, involving 2,585 and 27,591 unique miRNA and genes, respectively. Each interaction is assigned an integrative score, which is statistically inferred using predictions obtained from 30 independent resources^9^. The mirDIP database contains predicted scores for approximately 60% of all possible interactions for this set of miRNA and genes. Notably, the size of the predicted interaction set for a given miRNA varies within the range [44, 17596] with an average of 15,055 and median of 15,118. The size of the gene set varies within the range [5, 2375] with an average of 2,207 and median of 2,375 unique miRNAs targeting a typical gene.

### Experimentally validated miRNA–target interactions

Two sets of experimentally validated miRNA–gene interactions were obtained from DIANA-TarBase v8^7^ and mirTarBase v.8.0. The number of validated interactions between these databases is 684,107, consisting of 2,585 miRNAs and 17,629 genes. We eliminated any genes lacking at least one validated interaction; the O2A, by definition, requires at least one validated data point to extract the context-based features.

These validated interactions were then split into a training set for the cascaded classifier and a hold-out test set for independent performance evaluation. In order to mitigate potential bias by using test data that may have also been used to train mirDIP or its sources, the test set was curated by selecting only recently validated interactions. Recency was enforced by collecting a test set comprised only of interactions new to TarBase v8, which was published after the acceptance of the mirDIP 4.1 publication^9^. The resulting test set comprised 31,131 positively validated interactions; the remaining 652,976 positive samples were used for training the RP model.

To train and test the RP model, a set of negative pairs is also required. This poses a challenge, as there is a lack of validated non-interactions. Among published non-interactions, the reliability of these findings is considered by some to be questionable^9^. Furthermore, Helwak and Tollervey emphasized that pair non-interactivity may be due to properties of the specific detection method used rather than a property of the pair interactivity itself^12^. To overcome this, we conservatively assumed that pairings that are not validated are non-interactions. A uniform, random sample of these pairs was obtained to match the number of validated interactions in order to create a balanced training and testing set. Since the vast majority of miRNA–gene pairs are not expected to interact, selecting our negative set in this way was reasonable and follows the methodology used in several previous studies^9,27^.

### Adapting the baseline estimation for variable and sparse one-to-all curves

The O2A curve plots the rank-order distribution of scores involving a given miRNA or a given gene. That is, if a given miRNA has a predicted score for 200 distinct genes, its O2A is a monotonically decreasing curve with 200 points; the highest scoring gene is plotted with rank 1, the second highest as rank 2, and so on. In its original formulation, the RP method would require that every miRNA be scored against all possible genes, and conversely, all genes be scored against all possible miRNAs^26^.

A consequence to relaxing the complete-graph constraint is a variable number of data points within the rank-order O2A for a given miRNA or gene. For example, a given gene that has only been predicted to interact with ten miRNAs will have a sparse O2A as compared to an miRNA that has been predicted to target hundreds of genes. The original baseline estimation method used within RP leveraged LOESS to first fit a smooth curve to the O2A data. This approach assumed a sufficiently large number of data points to reliably identify the knee of the curve. Therefore, for curves with a sufficient number of points (i.e. $n > 100$), the Kneedle algorithm^28^ was used to locate the knee of the curve. To accommodate those curves with sparse number of points (i.e. $1 \le n \le 100$), which only represented less than 1% of the curves, we leveraged the median of the distribution of scores in the O2A to define the baseline.

**Table 1:** Extracted RP features for use in the cascaded model.

**Feature name**	**Feature details**		
	Short name	*Type*	*Description*
Percentile-XY	*rxy*	Rank	Rank order of gene Y among all the predictions for miRNA X
Percentile-YX	*ryx*	Rank	Rank order of miRNA X among all the predictions for gene Y
Adjusted reciprocal rank order	*ARRO*	Rank	Reciprocal product of *rxy* and *ryx*
Percentile-local-cutoff-X	*rxt*	Rank	Percentile rank of the gene nearest to the local cutoff value of miRNA X
Score-Local-Cutoff-X	*sxt*	Score	Score at the local cutoff value of miRNA X
Percentile-local-cutoff-Y	*ryt*	Rank	Percentile rank of the miRNA nearest to the local cutoff value of gene Y
Score-Local-Cutoff-Y	*syt*	Score	Score at the local cutoff value of gene Y
Percentile-difference-from-local-X	*pdx*	Fold	Difference between *rxy* and *rxt*
Percentile-difference-from-local-Y	*pdy*	Fold	Difference between *ryx* and *ryt*
Fold-difference-from-local-X	*fdx*	Fold	As defined in^26^
Fold-difference-from-local-Y	*fdy*	Fold	As defined in^26^
SD-distance-from-mean-X	*Stdx*	Stats	The number of standard deviations from the mean score in miRNA X
SD-distance-from-mean-Y	*Stdy*	Stats	The number of standard deviations from the mean score in gene Y

### Adaptation of RP feature calculation

For each miRNA–gene interaction, a set of features adapted from the original RP implementation were computed based on the identified baseline. These features leverage the distribution of non-validated pairs to extract “context”-based information available from all the scored interactions involving a specific miRNA and gene.

Modifications to the original RP implementation enabled its application to the miRNA–gene task. To accommodate a variable number of data points in the O2A, we substituted the absolute rank order of a scored pair in favour of its percentile rank. This ensured that rank-type features could be compared across different sized miRNA and gene perspectives. The binary RP metric “Above-Global-Threshold” was excluded as it was determined that RP models could learn the optimal global threshold using the raw mirDIP score. Similarly, the binary features for indicating if a score is above a local threshold were excluded as the Fold-Difference metrics provide similar information to the model. The resulting features used for training the cascaded learning algorithm are shown in Table 1.

### Training the cascaded machine learning model

Two learning algorithms were independently considered for the training and evaluation of the RP cascaded model. From its reported successes as part of the cascaded RFMirTarget method, we considered a Random Forest model. The model, denoted RPmirDIP*, was trained on the RP features derived from the training set (Table 1). Ten-fold cross validation was performed for hyperparameter tuning. This produced a model where the maximum size of the feature subset considered at each split was four, the forest comprised 100 trees, each with a maximum tree depth of 19. For its widespread application in various machine learning tasks and reported success within Kaggle competitions, the eXtreme Gradient Boosting (XGBoost) model was also considered. The model, denoted RPmirDIP, was trained on the RP features derived from the training set (Table 1). The learning task used logistic regression as the objective function and ROC AUC as the evaluation metric. The default parameters for the tree booster were used: the learning rate was set to 0.3, the $\gamma$ parameter was set to 0, and the max depth was set to 6. The model was trained over 200 iterations with an early stopping of five rounds.

### Evaluating RP performance improvement using independent miRNA targeting predictors

To comprehensively evaluate the utility of RP for improving the predictive performance for independent miRNA targeting predictors, we compared the performance of the predictor alone with the RP-augmented predictions of that predictor, mirDIP, and RPmirDIP. Twenty-six independent methods were augmenting using the RP cascaded model. For each method and its dataset of pre-computed predictions, RP features were calculated (Table 1). Since each individual dataset did not contain predictions for the entirety of the training and testing set, only the overlapping subset was used (Supplementary Figure 2). We note that the training datasets used to produce the predicted scores for each individual method in the mirDIP database are unknown which risks the possible inclusion of training samples among these datasets. This may result in the possible overstatement of results, however, this bias would be consistent across the four compared models which makes for a fair comparison. For each method, the XGBoost algorithm, using the same hyperparameters as RPmirDIP, was trained using the corresponding training set and features. To evaluate performance, the testing set was used to calculate the ROC AUC and PR AUC.

## Results and Discussion

Of the two learning algorithms independently trained on the RP features, Random Forest and XGBoost, the most performant was selected as the official RPmirDIP model (XGBoost) while the less performant was denoted the RPmirDIP* model (Random Forest). Here, we report and discuss the results of the RPmirDIP model and leave the RPmirDIP* results to the Supplementary Materials. Similarly, the experiments applying RP to 26 individual miRNA targeting predicitors are sumarized with supportive material in the Suppmentary Materials.

**Figure 3:**
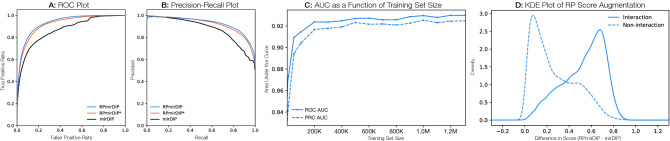
Results comparing the predictive performance of RPmirDIP and RPmirDIP* models against the original mirDIP predictions. (**A**) and (**B**) depict the ROC and PR curves, respectively. (**C**) illustrates the change in predictive performance as a function of the size of the training set. (**D**) is a kernel density estimation plot of the pair-wise difference in scores between RPmirDIP and mirDIP.

**Figure 4:**
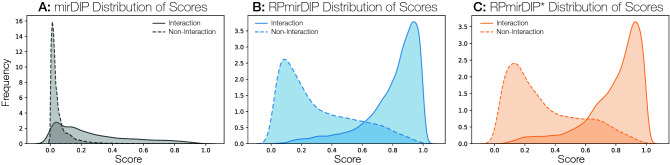
Comparison of the distribution of scores between mirDIP (**A**), RPmirDIP (**B**), and RPmirDIP* (**C**).

The evaluation of the performance difference between the original mirDIP method and the RP-augmented scores was achieved using bootstrap testing ($n = 1,000$) on the test set choosing both the area under the resulting precision-recall and receiver operating characteristic curves (PR AUC and ROC AUC, respectively). Considering the null hypothesis, ($H_0$: no significant difference in AUC between Original mirDIP and RP-augmented mirDIP), *p* values were computed using Welch’s unequal variances *t*-test and the observed differences in AUC were significant at the $p < 0.001$ level (Table 2; Fig. 3A,B). Interestingly, the RPmirDIP model benefited considerably from an increasingly large training set, with sharp improvements in AUC observed as the training set size approached 100K samples with relatively diminishing, though marked, improvement thereafter (Fig. 3C). The distributions of mirDIP, RPmirDIP, and RPmirDIP* scores are depicted in Fig. 4.

**Table 2:** Performance following 1,000 bootstrap iterations. Bold identifies the highest score achieved by a model for a given metric.

Predictor	Performance metric ($\mu \pm \sigma$)	
	ROC AUC	PR AUC
mirDIP	$0.8666 \pm 0.0015$	$0.8769 \pm 0.0017$
RPmirDIP*	$0.9210 \pm 0.0011$	$0.9198 \pm 0.0013$
RPmirDIP	$\mathbf {0.9311} \pm \mathbf {0.0009}$	$\mathbf {0.9262} \pm \mathbf {0.0014}$

**Figure 5:**
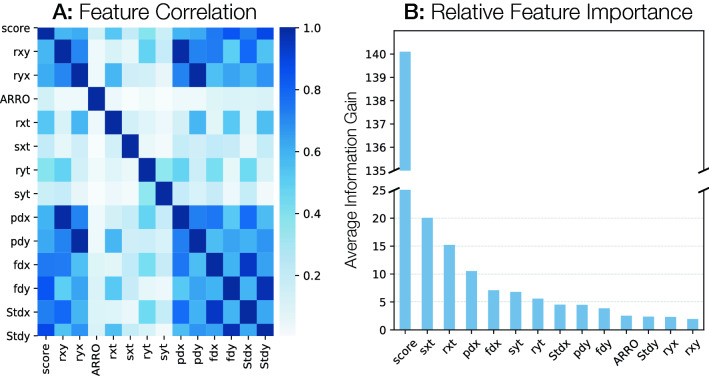
Exploration of RPmirDIP Features. (**A**) is a heatmap of feature correlation. Notably, the ARRO metric is the least correlated with other features and, as expected, rank (*rxy*, *ryx*) are strongly correlated with percent difference (*pdx*, *pdy*), respectively. (**B**) Plots the gain of a given feature as indicative of the relative contribution of that feature to the model based upon the feature’s contribution to each tree in the model. Note the break in the *y*-axis due to the large relative information gain on the initial score. Formally, gain is the average reduction in training loss when selecting that feature for splitting.

A further analysis of the relative feature importance from the resultant RPmirDIP model revealed a heavy reliance upon the raw mirDIP score, which is intuitive given that RPmirDIP needed to compensate for the removal of the binary “Above-Global-Threshold” feature which originally captured global-level information (Fig. 5). Notably, the RP-derived features, particularly those from the miRNA persepctive (*sxt*, *rxt*, *pdx*, *fdx*), each contributed complimentary information to the model, exemplified both by their low correlation with the mirDIP score and relatively large information gain. Furthermore, while the RPmirDIP model does not place considerable emphasis on the *ARRO* feature which has the lowest correlation with the mirDIP score, the RPmirDIP* model considers it as the third highest feature by importance (Supplementary Fig. 1A). This strongly suggests that the ARRO feature, which encodes the reciprocal, context-based information provided from both perspectives, is independent of the predicted score and provides complimentary information useful to distinguishing interacting pairs from non-interacting pairs.

We further analyzed the RP contribution to increased performance by computing the score augmentation when applying RPmirDIP and plotting the distribution of the score difference with mirDIP for the validated interactions and non-interactions from the test set (Fig. 3D). Interestingly, we observe a consistently positive increase in score for both classes with only a rare few receiving a decreased score. Consistent with the strong results observed for RMmirDIP, the validated interactions generally received a larger score augmentation than the non-interacting samples. These findings suggest that the use of interaction-specific context-based RP features increase overall predictive performance by augmenting the scores of true interactions for which the initial predictors had originally assigned a lower score that failed to exceed the globally defined threshold. This further suggests that the application of RP leverages previously underutilised information within pair-wise data sets to increase discriminability of the classes.

### Reciprocal Perspective improves performance of 26 miRNA targeting predictors

**Figure 6:**
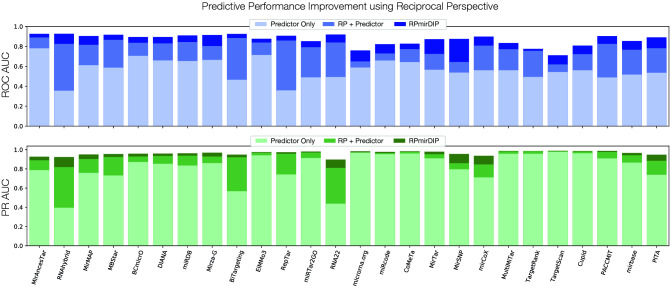
Increase in individual predictor performance using RP and the RPmirDIP model. The 26 prediction methods are ordered by the size of the available training set and each is notably improved with the combined use of RP and the use of the ensembled mirDIP score leveraged by RPmirDIP.

To comprehensively determine whether RP would consistently improve the predictive performance of independent miRNA targeting predictors regardless of their unique architectures and implementations, we compared the predictor’s performance with an RP-augmented model as well as the mirDIP and RPmirDIP models (Fig. 6). Promisingly, in all cases the cascaded application of RP to each predictor resulted in a notable increase in ROC AUC (between [0.0606–0.4999]) and PR AUC (between [0.0045–0.4333]). Comparing the RPmirDIP model trained on the subset of data available to each method (see Supplementary Materials for details), we observed further increases in ROC AUC (between [0.0198–0.2321]) as well as PR AUC (between [0.0021–0.1048]). Table 3 summarizes the various sizes of the datasets used for each experiment and supporting information can be found in the Supplementary Materials.

We note that where the initial predictor performs particularly well, there is little additional performance gain to be made (e.g. PR AUC of TargetRank and TargetScan). Conversely, where the initial model performs relatively poorly, there are substantial gains in performance observed, both in the application of RP to that model as well as with the use of the ensembled mirDIP score in conjunction with RP (i.e. RPmirDIP). From these experiments, we arrive to the following conclusions:

1. The cascaded application of RP to an initial predictor results in improved predictive performance (i.e. RP + Predictor).
2. The cascaded application of RP to an ensemble-based predictor (e.g. mirDIP) may result in further improvement in predictive performance (i.e. RPmirDIP).
3. RP compliments miRNA targeting predictor reliant only on the scores produced by that predictor and no other information.

**Table 3:**
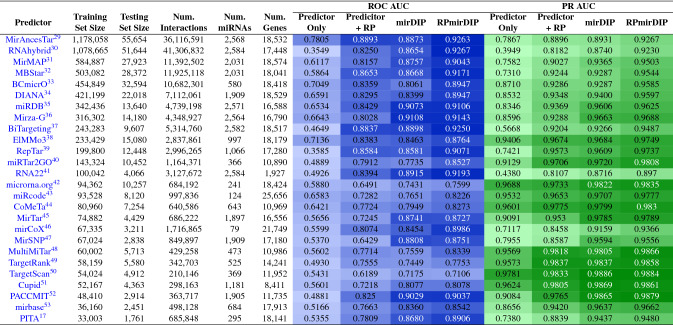
Comparison of the predictive performance of 26 individual miRNA targeting predictors.

### Discovering novel putative interactions

The accurate prediction of miRNA–target interactions is critical to our understanding of dynamic biological regulation networks. In miRNA–target prediction, RPmirDIP represents a novel method to discover new and possibly unexpected interactions that current predictors missed. Analyses and experimental validation are warranted for the set of interaction which mirDIP originally assigned low confidence scores, in contradiction to RPmirDIP’s assignment of a substantially higher score. These putative interactions promise potentially unique information that may improve our overall understanding of miRNA targeting and gene expression networks. For the benefit of the broader scientific community, we make available the sorted list of these putative interactions, available at 10.5683/SP2/LD8JKJ^54^.

We re-scored all $\sim \! 30$ million pairs listed in the mirDIP database using both the RPmirDIP and RPmirDIP* models. We then used the RPmirDIP scores to compute the Difference of Scores (DoS) with the original mirDIP prediction, defined as the RPmirDIP score minus the mirDIP score. With a noticeable peak in non-interactors around a DoS of 0.5 in Fig. 3D, we posit that these pertain to putative false negatives. Of the $\sim \! 30$ million predictions, we extracted those with a $DoS \ge 0.5$ and then sorted this set in two ways, one by DoS and another by RPmirDIP score; the top-10 interactions for each set are tabulated in Table 4. An analogous table of the top-10 interactions stored by mirDIP score is available in the Supplementary Materials. Following the mirDIP dataset convention, each of these sets were split into top-1%, top-5%, top-10%, top-33%, and bottom-66% sets and were released to the scientific community. The data are available at cu-bic.ca/RPmirDIP and 10.5683/SP2/LD8JKJ,^54^.

**Table 4:** Top-10 predictions when sorted by difference of score (top) and by RPmirDIP Score (bottom). Bold emphasizes the column that is sorted.

Top-*k* rank	miRNA	Gene	Difference in score	RPmirDIP score	mirDIP score
1	hsa-miR-522-5p	AGO2	**0.9798**	0.9868	0.0070
2	hsa-miR-522-5p	ANXA4	**0.9773**	0.9809	0.0036
3	hsa-miR-522-5p	TSC22D2	**0.9764**	0.9813	0.0048
4	hsa-miR-522-5p	FBXO33	**0.9743**	0.9785	0.0042
5	hsa-miR-522-5p	NCKAP1	**0.9735**	0.9776	0.0041
6	hsa-miR-522-5p	EIF4A2	**0.9728**	0.9772	0.0043
7	hsa-miR-522-5p	CASTOR2	**0.9695**	0.9732	0.0036
8	hsa-miR-34a-5p	SLC10A6	**0.9690**	0.9764	0.0074
9	hsa-miR-522-5p	MARCKS	**0.9688**	0.9724	0.0036
10	hsa-miR-522-5p	MAPK6	**0.9679**	0.9718	0.0039
1	hsa-miR-34a-5p	PKNOX1	0.5053	**0.9949**	0.4895
2	hsa-miR-34a-5p	WDR37	0.5394	**0.9926**	0.4532
3	hsa-miR-34a-5p	TUBB2A	0.8758	**0.9925**	0.1167
4	hsa-miR-34a-5p	GRSF1	0.5112	**0.9924**	0.4812
5	hsa-miR-34a-5p	OTUD3	0.5125	**0.9919**	0.4793
6	hsa-miR-34a-5p	CALM3	0.6151	**0.9918**	0.3768
7	hsa-miR-675-5p	LRIG2	0.5201	**0.9916**	0.4716
8	hsa-miR-16-5p	ALYREF	0.7314	**0.9916**	0.2602
9	hsa-miR-34a-5p	SNX30	0.5229	**0.9915**	0.4685
10	hsa-miR-34a-5p	PALM2	0.5333	**0.9913**	0.4580

The two sorting methods are meant to capture differing and complimentary rankings of interactions. The results sorted by RPmirDIP score are analogous to the results presented in the mirDIP database; they correspond to those interactions for which the trained model places the greatest confidence in being a true interaction. Sorted in this way, the ranking doesn’t account for the magnitude of the DoS; pairs high-scoring in mirDIP can be expected to be generally high-scoring in RPmirDIP.

The results sorted by DoS present pairs that are of a possibly more serendipitous nature. That is, these results are those for which mirDIP assigned very little confidence (possibly considered to be non-interacting) yet RPmirDIP assigned great confidence. Per the KDE plot in Fig. 3D, these are the pairs with the greatest likelihood of being novel discoveries to elucidate new gene regulatory dynamics. By definition, the predicted pairs with the highest DoS also tend to have very high RPmirDIP scores. While not included within this work, there also exists the possibility to generate a new ranking of pairs by combining scores. For example, generating a ranking for which *both* mirDIP and RPmirDIP are most confident, the mirDIP score, $m_i$, of a given pair *i* can be multiplied by the RPmirDIP score, $r_i$. Applied to all pairs, the resulting set of scores, ${\varvec{s}}$, can be sorted into rank order (and optionally min-max normalized to reside in [0,1]):

1 $$\begin{aligned} s_i = m_ir_i \end{aligned}$$

This assumes an equal weight (i.e. 0.5) is given to both the mirDIP and RPmirDIP scores. A generalization of this transformation would incorporate a weighting factor, $\lambda \in [0,1]$, for the RPmirDIP score such that the mirDIP score is weighted by a factor $1-\lambda$:

2 $$\begin{aligned} s^{\lambda }_{i} = (1-\lambda )m_i \times \lambda r_i \end{aligned}$$

For example, more heavily weighting the RPmirDIP score using $\lambda =0.9$ yields the set:

3 $$\begin{aligned} \varvec{s^{0.9}} ~\leftarrow ~ s^{0.9}_{i} = 0.1 m_i \times 0.9 r_i,~~\forall ~i \end{aligned}$$

Moreover, this form of exploratory re-ranking can be applied between any of the three available values, RPmirDIP score, mirDIP score, and the DoS. However, the multiplication of either RPmirDIP and DoS or mirDIP and DoS with a $\lambda =0.5$ will produce the identical ranking as the ranking by DoS, by definition. The investigation of such rankings is left to the users of the RPmirDIP datasets and future work.

### Review of corroborating literature

Considering the twenty interactions resulting from the top-10 pairs sorted by RPmirDIP score and DoS, we reviewed the existing literature for corroborating evidence. We summarized relevant details of the four miRNAs appearing within this set in Table 5 along with selected references. Interestingly, each of the four appear to have critical oncogenic roles, typically in the suppression of factors leading to cell proliferation and metastasis; three of the four miRNAs are involved in the suppression of non-small cell lung cancer proliferation and metastasis. Specifics to each miRNA and their predicted mRNA partners are discussed in the following sections. The corresponding Table 4 results produced from the RPmirDIP* model are available in Supplementary Table 1.

**Table 5:** Details of the miRNAs identified among the Top-10 RPmirDIP predictions.

miRNA	Accession	Sequence	Selected sources
hsa-miR-522-5p	MIMAT0005451	16-cucuagagggaagcgcuuucug-37	^55,56^
hsa-miR-34a-5p	MIMAT0000255	22-uggcagugucuuagcugguugu-43	^57–59^
hsa-miR-675-5p	MIMAT0004284	10-uggugcggagagggcccacagug-32	^60,61^
hsa-miR-16-5p	MIMAT0000069	14-uagcagcacguaaauauuggcg-35	^62,63^

#### hsa-miR-522-5p

Among the set of top-10 predicted pairs sorted by DoS, hsa-miR-522-5p is involved in nine. While the mirDIP dataset assigns a nearly negligible score to each of these pairs, the RPmirDIP score approaches the maximal value producing the highest possible DoSs. Interestingly the mirDIP dataset lists 18,537 scores involving hsa-miR-522-5p however only four genes are ranked within the “Very High” confidence class:

1. Gene: NRCAM, Accession: Q92823, mirDIP: 0.4338
2. Gene: CBX4, Accession: O00257, mirDIP: 0.4047
3. Gene: CBX8, Accession: Q9HC52, mirDIP: 0.3941
4. Gene: GIPC3, Accession: Q8TF64, mirDIP: 0.3863

Each of the nine genes fell within the bottom 66% or “Low” confidence class. Furthermore, the RNACentral entry for this miRNA lists 2,964 interacting target genes and their proteins, however, none of which are the nine among these top-10, suggestive that these nine interactions may present novel interactions. RNACentral does list targeted genes related to those predicted by RPmirDIP: the Annexin protein ANXA5 is targeted, but not the predicted ANXA4; the F-Box proteins FBXO9, FBXO25, FBXO32, and FBXO45 are each targeted, but not the predicted FBXO33; the Eukaryotic transition initiation factor 4 (EIF4) proteins EIF4G1, EIF4G2, EIF4B, EIF4EBP2, and EIF4EBP3 are each targeted, but not the predicted EIF4A2; finally, the Mitogen-Activated Protein Kinase (MAPK) proteins MAPK1IP1L, MAPKAPK3, MAPK14, MAPKAPK2, and MAPK9 proteins are each targeted, but not the predicted MAPK6. No proteins related to any of AGO2, TSC22D2, NCKAP1, CASTOR2, or MARCKS were listed in RNACentral.

Of the nine candidate interactors, AGO2 is an effector of small RNA mediated gene silencing and, therefore, a promising candidate interactor with critical roles in oncogene progression^64^. FBXO33 is a prognostic marker in both renal and lung cancers, while CASTOR2 is disease-associated with spinal cord glioma, spinal cancer, and its RNA is overexpressed in testis cancer. The miRBase dataset lists 16 open access papers that mention hsa-mir-522, many relating to the proliferation of tumor cells. The miRNA, when downregulated, suppresses tumorigenesis by directly regulating the DENN Domain Containing 2D (DENND2D) tumor suppression gene for non-small cell lung cancer cells^55^. Additionally, hsa-miR-522-5p regulates cell proliferation, detachment, migration, and the epithelial-mesenchymal transition^56^. This curated evidence, while preliminary and circumstantial, suggests that hsa-miR-522-5p may have a more extensive role in cancer dynamics, warranting further investigation.

#### hsa-miR-34a-5p

The hsa-miR-34a miRNA is a regulator of tumor suppression and its use as part of an miRNA-based oncosuppressor replacement therapy is an effective strategy against tumor heterogeneity^59^. Among the set of top-10 predicted pairs sorted by RPmirDIP score, hsa-miR-34a-5p is involved in eight, with a ninth among the top-10 by DoS. The mirDIP database lists 19,748 pairs involving hsa-miR-34a-5p of which 1,330 are within the “Very High” confidence class, containing six of the nine genes (excluding TUBB2A, CALM3, and SLC10A6). Therefore, these predictions may may simply reiterate previous findings from an miRNA having been previously extensively investigated, reinforcing previous findings. To that point, the miRBase database lists 927 open access papers that mention hsa-mir-34a. Numerous studies demonstrate hsa-mir-34a’s involvement in the initiation and progression of cancers. It has been demonstrated to inhibit the proliferation and metastasis of osteosarcoma cells both in vitro and in vivo^57^. The under-expression of hsa-mir-34a led to the development and progression of human malignancy via Notch1^58^. Moreover, in combination with p53, hsa-miR-34a-5p has been demonstrated to suppress colorectal cancer metastasis by inhibiting cell proliferation, migration, and invasion^65^.

While not directly implicated in the development or progression of human cancers, we here briefly introduce the major functions associated to the predicted pairs. To appreciate the diversity of genes and functions highly predicted with this miRNA, the analyses should be expanded beyond the top-10. The Solute Carrier Family 10 Member 7 (SLC10A7) protein was targeted in the RNACentral entry of hsa-miR-34a-5p, but the predicted SLC10A6 was not. This family of proteins is responsible for the sodium-dependent transport of sulfoconjugated steroid hormones, taurolithocholic acid-3-sulfates, sulfoconjugated pyrenes^66^. The PKNOX1 is a homeobox gene that is disease-associated with Down Syndrome^67^. Members of the WD repeat protein family are involved in a variety of cellular processes such as apoptosis, cell cycle progression, gene regulation, and signal transduction^68^. The G-rich sequence factor 1 (GRSF1) protein regulates post-transcriptional mitochondrial gene expression and is necessary to recruit mRNAs to mitochondial ribosomes. The OTU domain-containing protein 3 (OTUD3) hydrolyzes Lys-6- and Lys-11-linked polyubiquitin. The Calmodulin-3 (CALM3) protein mediates the activity of a diverse array of proteins via calcium-binding. The Sorting nexin-33 (SNX30) protein is required to eficiently progress through mitosis and cytokinesis (e.g. necessary for normal formation of the cleavage furrow at the end of mitosis). Finally, the Paralemmin-2 (PALM2) protein is disease-associated with Hypertrichosis Universalis Congenita, Ambras Type and Kallmann Syndrome.

#### hsa-miR-675-5p

The hsa-miR-675-5p miRNA is another that has been extensively studied for its role in suppressing cancers, it has been found overexpressed in many cancers. The downregulation of miR-675-5p has previously been demonstrated to suppress lung cancer progression and metastasis through the regulation of the G protein-coupled receptor 55 (GPR55)^60^. Another study evidences its oncogenic role in esophageal squamous cell carcinoma (ESCC) by inhibiting RALBP1 Associated Eps Domain Containing 2 (RESP2) via the RalBP1/RAC1/CDC42 signaling pathway; among ESCC patients, hsa-miR-675-5p is a valuable prognostic biomarker and therapeutic target^61^. The miRBase database lists 106 open access papers that mention hsa-mir-675.

One pair involving this miRNA appeared among the top-10, involving the Leucine-Rich Repeats and Immunoglobulin-Like Domains 2 (LRIG2) gene. The mirDIP database lists LRIG2 as the 5th gene among the top-28 “Very High” confidence interactions within the mirDIP database. The encoded LRIG2 protein is known to promote epidermal growth factor signalling leading to increased cell proliferation. Promisingly, this function suggests that miR-676-5p regulation of the LRIG2 gene would suppress proliferation, as seen in the miRNA’s related activity. Our findings suggest that wet laboratory investigations into hsa-miR-675-5p and LRIG2 interactions are warranted.

#### hsa-miR-16-5p

The hsa-miR-16-5p miRNA is the last among the top-10 predicted pairs. It is another tumor suppressor and recently identified as a promising biomarker or therapeutic target for cholangiocarcinoma through its direct targeting of the yes-associated protein 1 (YAP1) transcriptional regulator^62^. Within breast cancer tumors, it is a stably-expressed housekeeping miRNA, found to be the most consistently expressed among other housekeeper candidate subtypes^63^. The miRBase database lists 730 open access papers that mention hsa-mir-16-1, which comprises hsa-miR-16-5p and hsa-miR-16-1-3p.

One pair involving this miRNA appeared among the top-10 predicted pairs, involving the Aly/REF export factor gene (ALYREF) that encodes the THO complex subunit 4 (THOC4) protein. RNACentral lists 4,821 target proteins although the THOC4 protein is not listed among them. The mirDIP classifies this pair among the “High” confidence class. Considering THOC4’s role as a prognostic marker in liver cancer and its detected expression in several cancers, this predicted pair is a likely candidate to play a more extensive role in oncogenic regulatory dynamics and warrants further wet laboratory investigation.

## Conclusion

This work demonstrates the successful application of the RP method to miRNA–gene prediction resulting in significantly improved predictive performance over mirDIP ($p < 0.001$). We present a pragmatic implementation of RP which relaxes the constraint for a complete CPM by leveraging pre-computed scores. Future work will investigate the impact of CPM (in)completeness as a function of improved predictive performance and score augmentation. We anticipate that the RPmirDIP method might be applied widely to miRNA–gene prediction and yield promising putative interactions which may form the basis of testable hypotheses. We made publicly available the set of the most likely candidates, available at 10.5683/SP2/LD8JKJ,^54^.

## Supplementary information

Supplementary information

## References

[1] Lu J (2005). Microrna expression profiles classify human cancers. Nature.

[2] Kloosterman WP, Plasterk RH (2006). The diverse functions of micrornas in animal development and disease. Dev. Cell.

[3] Forster SC, Tate MD, Hertzog PJ (2015). Microrna as type i interferon-regulated transcripts and modulators of the innate immune response. Front. Immunol..

[4] Ren Z, Ambros VR (2015). Caenorhabditis elegans micrornas of the let-7 family act in innate immune response circuits and confer robust developmental timing against pathogen stress. Proc. Natl. Acad. Sci..

[5] Hollins SL, Cairns MJ (2016). MicroRNA: small RNA mediators of the brains genomic response to environmental stress. Prog. Neurobiol..

[6] Iwasaki YW (2013). Global microrna elevation by inducible exportin 5 regulates cell cycle entry. RNA.

[7] Karagkouni D (2018). DIANA-TarBase v8: a decade-long collection of experimentally supported miRNA-gene interactions. Nucleic Acids Res..

[8] Thomson DW, Bracken CP, Goodall GJ (2011). Experimental strategies for microRNA target identification. Nucleic Acids Res..

[9] Tokar T (2018). mirDIP 4.1—integrative database of human microRNA target predictions. Nucleic Acids Res..

[10] Chi SW, Zang JB, Mele A, Darnell RB (2009). Argonaute HITS-CLIP decodes microRNA–mRNA interaction maps. Nature.

[11] Hafner M (2010). PAR-CliP—a method to identify transcriptome-wide the binding sites of RNA binding proteins. J. Vis. Exp..

[12] Helwak A, Tollervey D (2014). Mapping the miRNA interactome by cross-linking ligation and sequencing of hybrids (CLASH). Nat. Protoc..

[13] Alles J (2019). An estimate of the total number of true human miRNAs. Nucleic Acids Res..

[14] Pertea M, Salzberg SL (2010). Between a chicken and a grape: estimating the number of human genes. Genome Biol..

[15] John B (2004). Human microRNA targets. PLoS Biol..

[16] Agarwal V, Bell GW, Nam J-W, Bartel DP (2015). Predicting effective microrna target sites in mammalian mRNAs. eLife.

[17] Kertesz M, Iovino N, Unnerstall U, Gaul U, Segal E (2007). The role of site accessibility in microRNA target recognition. Nat. Genet..

[18] Doench JG, Sharp PA (2004). Specificity of microRNA target selection in translational repression. Genes Dev..

[19] Tabas-Madrid D (2014). Improving miRNA–mRNA interaction predictions. BMC Genomics.

[20] Ding J, Li X, Hu H (2016). Tarpmir: a new approach for microrna target site prediction. Bioinformatics.

[21] Mendoza MR (2013). RFMirTarget: predicting human microRNA target genes with a random forest classifier. PLoS One.

[22] Liu W, Wang X (2019). Prediction of functional microRNA targets by integrative modeling of microRNA binding and target expression data. Genome Biol..

[23] Cheng S (2015). MiRTDL: a deep learning approach for miRNA target prediction. IEEE/ACM Trans. Comput. Biol. Bioinform..

[24] Wen M, Cong P, Zhang Z, Lu H, Li T (2018). Deepmirtar: a deep-learning approach for predicting human miRNA targets. Bioinformatics.

[25] Pla A, Zhong X, Rayner S (2018). miRAW: a deep learning-based approach to predict microRNA targets by analyzing whole microRNA transcripts. PLoS Comput. Biol..

[26] Dick K, Green JR (2018). Reciprocal perspective for improved protein–protein interaction prediction. Sci. Rep..

[27] Korfiati A (2015). Predicting human miRNA target genes using a novel computational intelligent framework. Inf. Sci..

[28] Satopaa, V., Albrecht, J., Irwin, D. & Raghavan, B. Finding a “kneedle” in a haystack: Detecting knee points in system behavior. In *2011 31st international conference on distributed computing systems workshops*, 166–171 (IEEE, 2011).

[29] Leclercq M, Diallo AB, Blanchette M (2017). Prediction of human miRNA target genes using computationally reconstructed ancestral mammalian sequences. Nucleic Acids Res..

[30] Rehmsmeier M, Steffen P, Höchsmann M, Giegerich R (2004). Fast and effective prediction of microRNA/target duplexes. RNA.

[31] Vejnar CE, Zdobnov EM (2012). MIRmap: comprehensive prediction of microRNA target repression strength. Nucleic Acids Res..

[32] Bandyopadhyay S, Ghosh D, Mitra R, Zhao Z (2015). MBSTAR: multiple instance learning for predicting specific functional binding sites in microRNA targets. Sci. Rep..

[33] Yue D, Guo M, Chen Y, Huang Y (2012). A Bayesian decision fusion approach for microRNA target prediction. BMC Genomics.

[34] Reczko M, Maragkakis M, Alexiou P, Grosse I, Hatzigeorgiou AG (2012). Functional microrna targets in protein coding sequences. Bioinformatics.

[35] Wong N, Wang X (2015). miRDB: an online resource for microrna target prediction and functional annotations. Nucleic Acids Res..

[36] Gumienny R, Zavolan M (2015). Accurate transcriptome-wide prediction of microrna targets and small interfering RNA off-targets with MIRZA-G. Nucleic Acids Res..

[37] Veksler-Lublinsky I, Shemer-Avni Y, Kedem K, Ziv-Ukelson M (2010). Gene bi-targeting by viral and human miRNAs. BMC Bioinform..

[38] Gaidatzis D, van Nimwegen E, Hausser J, Zavolan M (2007). Inference of miRNA targets using evolutionary conservation and pathway analysis. BMC Bioinform..

[39] Elefant N (2011). RepTar: a database of predicted cellular targets of host and viral miRNAs. Nucleic Acids Res..

[40] Latysheva NS, Babu MM (2016). Discovering and understanding oncogenic gene fusions through data intensive computational approaches. Nucleic Acids Res..

[41] Miranda KC (2006). A pattern-based method for the identification of microrna binding sites and their corresponding heteroduplexes. Cell.

[42] Betel D, Koppal A, Agius P, Sander C, Leslie C (2010). Comprehensive modeling of microRNA targets predicts functional non-conserved and non-canonical sites. Genome Biol..

[43] Jeggari A, Marks DS, Larsson E (2012). miRcode: a map of putative microRNA target sites in the long non-coding transcriptome. Bioinformatics.

[44] Gennarino VA (2012). Identification of microRNA-regulated gene networks by expression analysis of target genes. Genome Res..

[45] Hsu JB-K (2011). miRTar: an integrated system for identifying miRNA–target interactions in human. BMC Bioinform..

[46] Giles, C. B., Girija-Devi, R., Dozmorov, M. G. & Wren, J. D. mircox: a database of mirna-mrna expression correlations derived from rna-seq meta-analysis. In *BMC Bioinformatics*, vol. 14, S17 (BioMed Central, 2013). 10.1186/1471-2105-14-S14-S17 PMC3850996 24267917

[47] Liu C (2012). MirSNP, a database of polymorphisms altering miRNA target sites, identifies miRNA-related SNPs in GWAS SNPs and eQTLs. BMC Genomics.

[48] Mitra R, Bandyopadhyay S (2011). MultiMiTar: a novel multi objective optimization based miRNA-target prediction method. PLoS One.

[49] Nielsen CB (2007). Determinants of targeting by endogenous and exogenous microRNAs and siRNAs. RNA.

[50] Lewis BP, Shih I-H, Jones-Rhoades MW, Bartel DP, Burge CB (2003). Prediction of mammalian microRNA targets. Cell.

[51] Chiu H-S (2015). Cupid: simultaneous reconstruction of microRNA-target and ceRNA networks. Genome Res..

[52] Marín RM, Voellmy F, von Erlach T, Vaníček J (2012). Analysis of the accessibility of clip bound sites reveals that nucleation of the miRNA: mRNA pairing occurs preferentially at the 3-end of the seed match. RNA.

[53] Griffiths-Jones S (2004). The microrna registry. Nucleic Acids Res..

[54] Kyrollos, D. G., Reid, B., Dick, K. & Green, J. R. RPmirDIP predictions of $$\sim$$6 million miRNA–gene pairs 10.5683/SP2/LD8JKJ (2020).

[55] Zhang T (2016). Downregulation of miR-522 suppresses proliferation and metastasis of non-small cell lung cancer cells by directly targeting DENN/MADD domain containing 2D. Sci. Rep..

[56] Tan SM (2014). Sequencing of captive target transcripts identifies the network of regulated genes and functions of primate-specific miR-522. Cell Rep..

[57] Yan K (2012). MicroRNA-34a inhibits the proliferation and metastasis of osteosarcoma cells both in vitro and in vivo. PLoS One.

[58] Wang X-P (2017). MicroRNA-34a regulates liver regeneration and the development of liver cancer in rats by targeting notch signaling pathway. Oncotarget.

[59] Misso G (2014). Mir-34: a new weapon against cancer?. Mol. Ther. Nucleic Acids.

[60] He D (2015). Down-regulation of mir-675-5p contributes to tumor progression and development by targeting pro-tumorigenic gpr55 in non-small cell lung cancer. Mol. Cancer.

[61] Zhou Y-W (2016). mir-675-5p enhances tumorigenesis and metastasis of esophageal squamous cell carcinoma by targeting reps2. Oncotarget.

[62] Han S (2017). Suppression of mir-16 promotes tumor growth and metastasis through reversely regulating yap1 in human cholangiocarcinoma. Oncotarget.

[63] Rinnerthaler G (2016). mir-16-5p is a stably-expressed housekeeping microrna in breast cancer tissues from primary tumors and from metastatic sites. Int. J. Mol. Sci..

[64] Zhang H (2019). Acetylation of ago2 promotes cancer progression by increasing oncogenic mir-19b biogenesis. Oncogene.

[65] Shi H (2016). mir-34a inhibits the in vitro cell proliferation and migration in human esophageal cancer. Pathol. Res. Pract..

[66] Geyer J (2007). Cloning and functional characterization of human sodium-dependent organic anion transporter (slc10a6). J. Biol. Chem..

[67] Sánchez-Font MF, Bosch-Comas A, Gonzàlez-Duarte R, Marfany G (2003). Overexpression of fabp7 in down syndrome fetal brains is associated with pknox1 gene-dosage imbalance. Nucleic Acids Res..

[68] Reis LM (2019). De novo missense variants in wdr37 cause a severe multisystemic syndrome. Am. J. Hum. Genet..

