# Supplementary material for: RPmirDIP: Reciprocal Perspective improves miRNA targeting prediction
Source: Sci Rep. 2020 Jul 16;10:11770. doi: 10.1038/s41598-020-68251-4 (PMC7366700; doi:10.1038/s41598-020-68251-4)
Supplement: Supplementary file 1 — Supplementary information [file 41598_2020_68251_MOESM1_ESM.pdf]

Supplementary Material

RPmirDIP: Reciprocal Perspective Improves miRNA Targeting Prediction

Daniel G. Kyrollos[1], **Bradley Reid**[1], **Kevin Dick**[1,2], **and James R. Green**[1,2,*]

[1]Department of Systems & Computer Engineering, Carleton University, Ottawa, Canada
[2]Institute of Data Science, Carleton University, Ottawa, Canada
[*]jrgreen@sce.carleton.ca

ABSTRACT

This supplementary materials document contains additional information on the development, training, and results of the RPmirDIP* model as well as the experiments applying Reciprocal Persepctive to 26 additional predictors. Finally, it contains further details related to the formatting of the published predictions and the adherence to the original mirDIP conventions.

The RPmirDIP* Model

When selecting the machine learning algorithm to train the Reciprocal Perspective (RP) cascaded classifier, we chose two decision tree based models for their ease of hyperparameter tuning, interpretability, and complimentarity in ensemble techniques: bagging versus boosting. The Random Forest model is the prototypical bagging technique, were the variance of a model is reduced by considering a subset of samples and features to train several independent decision trees. Inferences are then made by passing an unseen feature vector to each tree which, in turn, generates its prediction and a vote among all trees is used to produce the final prediction. The hyperparameter tuning of this model, denoted RPmirDIP8, produced a model where the maximum size of the feature subset considered at each split was four, the forest comprised 100 trees, and each tree was limited to a maximum tree depth of 19.

Following a similar evaluation process as the RPmirDIP model, which implements the XGBoost learning algorithm, the model relative feature importance was examined (Supplementary Figure 1A) along with the change in AUC values based on the training dataset size (Supplementary Figure 1B) and the kernel density plot (KDE) for the difference in scores (DoS; defined as RPmirDIp*-mirDIP; depicted in Supplementary Figure 1C).

Notably, the RP derived “ARRO" feature , despite having the lowest correlation with the mirDIP score, had the third highest feature importance. This strongly suggests that the ARRO feature, which encodes the reciprocal, context-based information provided from both perspectives, is independent of the predicted score. Whereas the RPmirDIP predictor places a sizable relative importance on the *score* feature, the RPmirDIP* model appears to more uniformly leverage each of the features available to the model. Specifically, there appears to be less of a long-tailed distribution in Supplementary Figure 1A.

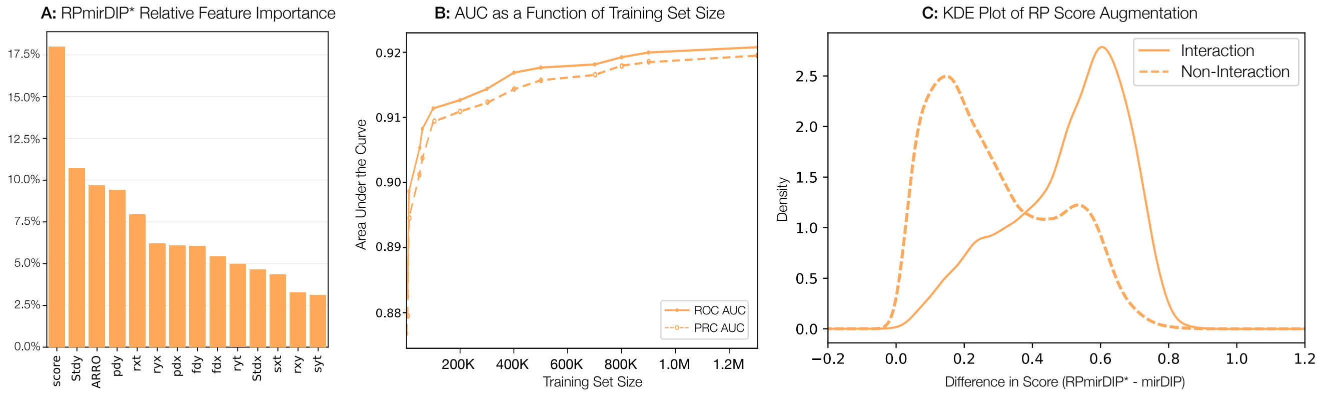

Supplementary Figure 1. Results of the RPmirDIP* Random Forest Model. (A) depicts the relative feature importance, (B) illustrates the monotonically increasing model performance based on the number of training samples used, (C) plots the augmentation in scores due to the application of RP within the RPmirDIP* model.

Supplementary Table 1. Top-10 Predictions when Sorted by Difference of Score (top) & by RPmirDIP* Score (bottom).

Top-k Rank	*miRNA*	*Gene*	*Difference in Score*	*RPmirDIP* Score*	*mirDIP Score*
1	hsa-miR-8485	CSF1	**0.9725**	0.9806	0.0081
2	hsa-miR-8485	KIAA0040	**0.9718**	0.9799	0.0081
3	hsa-miR-8485	DPYD	**0.9684**	0.9764	0.0081
4	hsa-miR-8485	EPB41	**0.9486**	0.9567	0.0081
5	hsa-miR-522-5p	FZD7	**0.9454**	0.9493	0.0039
6	hsa-miR-522-5p	HBEGF	**0.9453**	0.9492	0.0039
7	hsa-miR-522-5p	HOXA3	**0.9441**	0.9480	0.0039
8	hsa-miR-522-5p	HACD3	**0.9415**	0.9454	0.0039
9	hsa-miR-522-5p	FAM91A1	**0.9376**	0.9414	0.0038
10	hsa-miR-522-5p	SRSF12	**0.9348**	0.9386	0.0038
1	hsa-miR-146a-5p	KCNB2	0.9676	**0.9963**	0.02869
2	hsa-miR-146a-5p	CYSRT1	0.9672	**0.9958**	0.02867
3	hsa-miR-146a-5p	R3HCC1	0.9670	**0.9957**	0.02870
4	hsa-miR-146a-5p	PRSS38	0.9668	**0.9955**	0.02866
5	hsa-miR-146a-5p	TGM1	0.9666	**0.9953**	0.02866
6	hsa-miR-146a-5p	RNF208	0.9663	**0.9949**	0.02866
7	hsa-miR-146a-5p	ZP1	0.9660	**0.9947**	0.02870
8	hsa-miR-146a-5p	KRT7	0.9659	**0.9945**	0.02865
9	hsa-miR-146a-5p	LCE2B	0.9657	**0.9944**	0.02868
10	hsa-miR-146a-5p	PPY	0.9656	**0.9942**	0.02867

A complimentary analysis of the change in AUC values by training set size reveals a similar monotonically increasing trend to the RPmirDIP model (Supplementary Figure 1B). a notable increase in performance is observed when using 100K samples with a less drastic, yet marked increase thereafter. Promisingly, as databases continue to grow in size as the result of the exploration of additional miRNA-mRNA pairs with both wet laboratory and computation methods, the opportunity to leverage an even greater number of training samples should produce increasingly performant models.

The generation of a KDE plot from the DOSs between the predicted RPmirDIP* and mirDIP scores reveals a similar trend to that of the RPmirDIP model: the application of RP systematically increased the pair scores with a more exaggerated increase among the known interactors and a notable peak around $DoS \approx 0.5$ among the non-interactors. As mentioned in the main text, this peak may comprise possible false negatives and therefore warranting additional investigation by complimentary wet laboratory experimentation. To this end, the RPmirDIP* scores are also published along with the RPmirDIP scores for the benefit of the greater research community. Supplementary Table 1 lists the top-10 predicted pairs sorted by DoS (top) and RPmirDIP* score (bottom). These present only a minute fraction of all published predictions and a comprehensive analysis of these predictions is left to the benefit of the greater community as part of future work. While it initially appears that the top-10 predictions, sorted by RPmirDIP* score, appear to be sorted by both RPmirDIP* score *and* DoS, it is coincidental that the highest scoring pairs by RPmirDIP* score also represent pairs exhibiting the greatest RP-based augmentations in score. Finally, for completeness, we also highlight the top-10 interactions sorted by mirDIP score in Supplementary Table 2. We use an asterix to highlight those interactions that were previously known.

Applying RP to 26 Individual Predictors

To better understand whether the application of RP to individual predictors (in contrast to applying it to the mirDIP predictor alone which is an ensemble-based method), we obtained from the mirDIP dataset the subset of pairs for which a prediction score is available for a given method. This was repeated for the 26 methods in the mirDIP dataset which had at least 30,000 training pairs and at least 1,000 test pairs. Each method had a variable number of predicted scores requling in variable sized datasets for comparison. The relative differences in sizes are visualized in Supplementary Figure 2.

Interestingly, we observe a broad spectrum of dataset compositions, from the near-all-encompassing (*e.g.* MirAncesTar, RNAhybrid) to very limited (*e.g.* TargetRank, TargetScan). This diversity provides a rich experimental framework to evaluate the utility and robustness of of RP across a broad range of methods, dataset sizes, and compositions.

Supplementary Table 2. Top-10 Predictions when Sorted by Original mirDIP Score.

Top-k Rank	*miRNA*	*Gene*	*Difference in Score*	*RPmirDIP* Score*	*mirDIP Score*
1*	hsa-miR-22-3p	H3F3B	-0.0011	0.9844	**0.9855**
2*	hsa-miR-19b-3p	ZMYND11	-0.0047	0.9774	**0.9821**
3*	hsa-miR-19a-3p	ZMYND11	-0.0110	0.9707	**0.9817**
4*	hsa-miR-98-5p	BZW1	-0.0011	0.9900	**0.9811**
5*	hsa-let-7c-5p	BZW1	0.0122	0.9932	**0.9811**
6	hsa-miR-143-3p	LMO4	0.0061	0.9866	**0.9805**
7*	hsa-let-7b-5p	BZW1	0.0170	0.9971	**0.9801**
8*	hsa-let-7f-5p	BZW1	0.0173	0.9963	**0.9790**
9*	hsa-miR-145-5p	FLI1	-0.0329	0.9444	**0.9773**
10*	hsa-miR-29c-3p	PMP22	0.0053	0.9824	**0.9770**

* Indicates that this is a known interaction.

Promisingly, a substantial increase in performance from the application of RP is observed for all 26 methods. The ROC and PR curves for each method is provided in Supplementary Figures 3 and 4. Interestingly, the RP + Predictor performance, in a large number of instances, outperforms mirDIP alone. This suggests that the simple application of RP (that doesn't require any additional information beyond the predicted scores) to a single method improves the model to an equivalent, and possibly, superior performance as compared to an ensemble-based method (that requires considerable complimentary information).

Furthermore, for each of the 26 predictors, we plotted the distribution of scores of the original method, the distribution of scores from the application of RP, and the distribution of differences of scores (the original predictor score is subtracted from the RP+Predictor score). The results are presented in Supplementary Figures

Published Predictions

Finally, we wish to clarify the organization of the published data for use by the greater community. The published data are organized according to the the file format convention used by the mirDIP database. That is, the data are available for download in percentile sets where the top-*k*% of interactions are included in a single file, without duplication. For example, the file containing the top-10% of predictions only contains the 6-10th percentile, while the top-5% of predictions contain the 2-5th percentile, while the top-1% contains the 1st percentile. When accessing the top-10% data, it is therefore necessary to also select both the top-5% and top-1% files. An illustration of this convention with respect to the published files is depicted in Supplementary Figure 9.

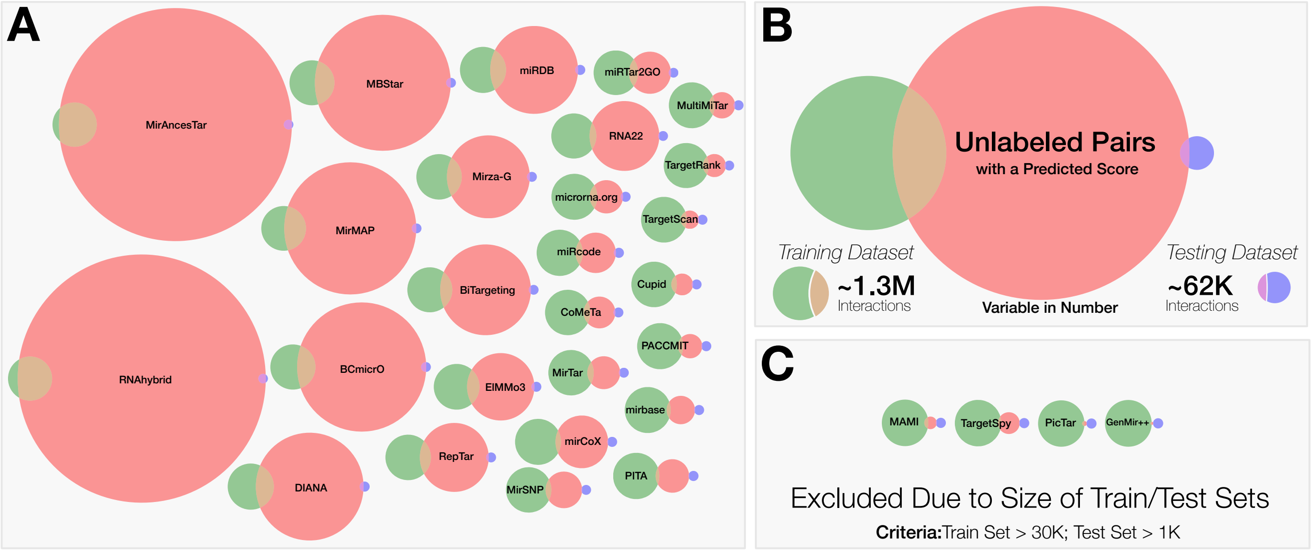

Supplementary Figure 2. Pictorial representation of the variable sizes of each dataset used for the evaluation of each of the 26 individual predictors. Panel A depicts a Venn diagram for each of the 26 methods considered roughly ordered from left to right and top-down by the size of the available training set for that method (yellow area). All diagrams are scaled such that the 1.3M training and 62K testing datasets are constant in size. Panel B is a legend illustrating the relationship between available scores for a given predictor with respect to the complete training and test set used throughout this study. Panel C highlights the four excluded methods for failure to meet our inclusion criteria.

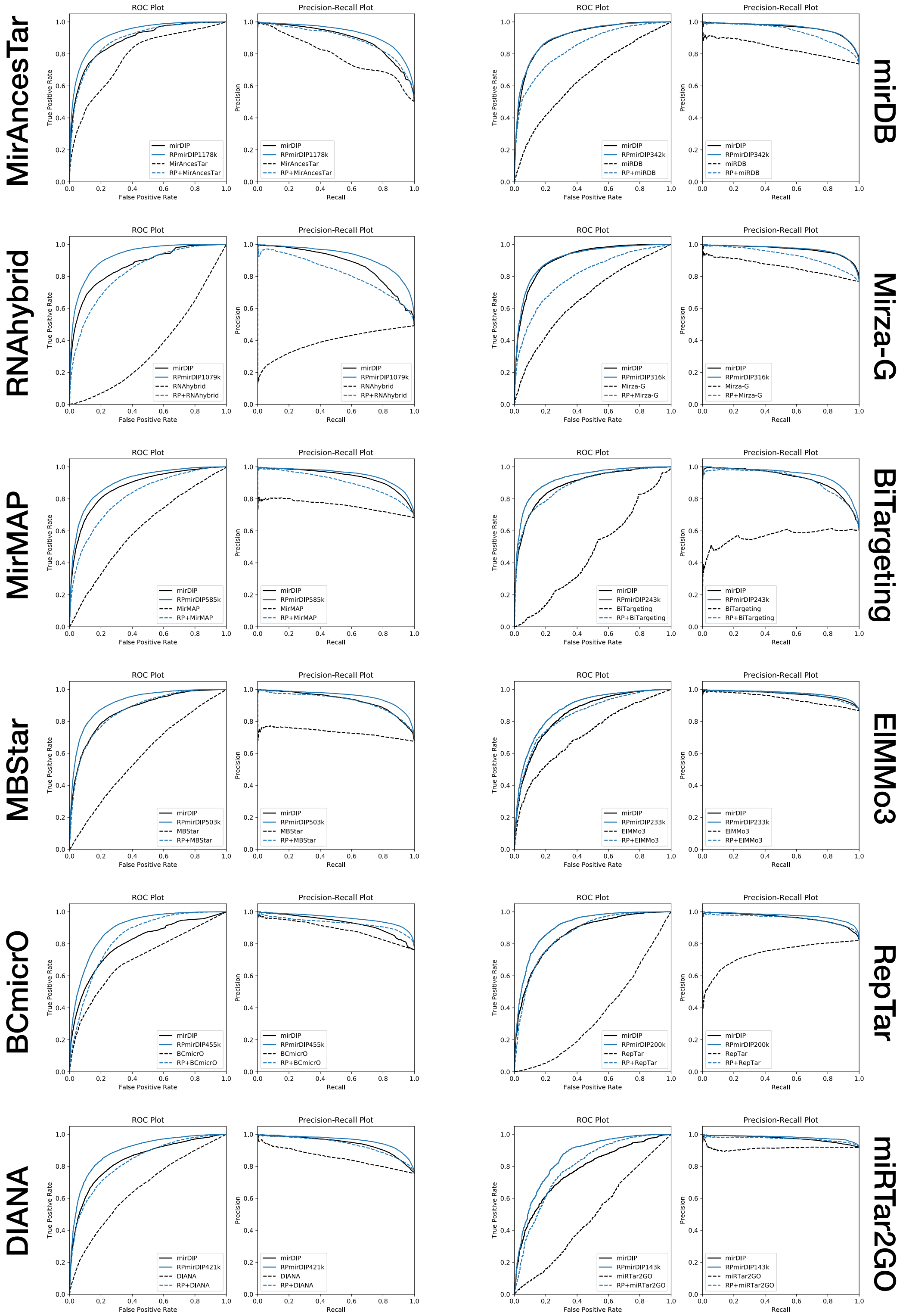

Supplementary Figure 3. ROC and PR curves for the top-12 predictors by training set size. To ensure a fair comparison, each of the RPmirDIP predictors compared against each model was only trained on the subset of score pairs available to each method.

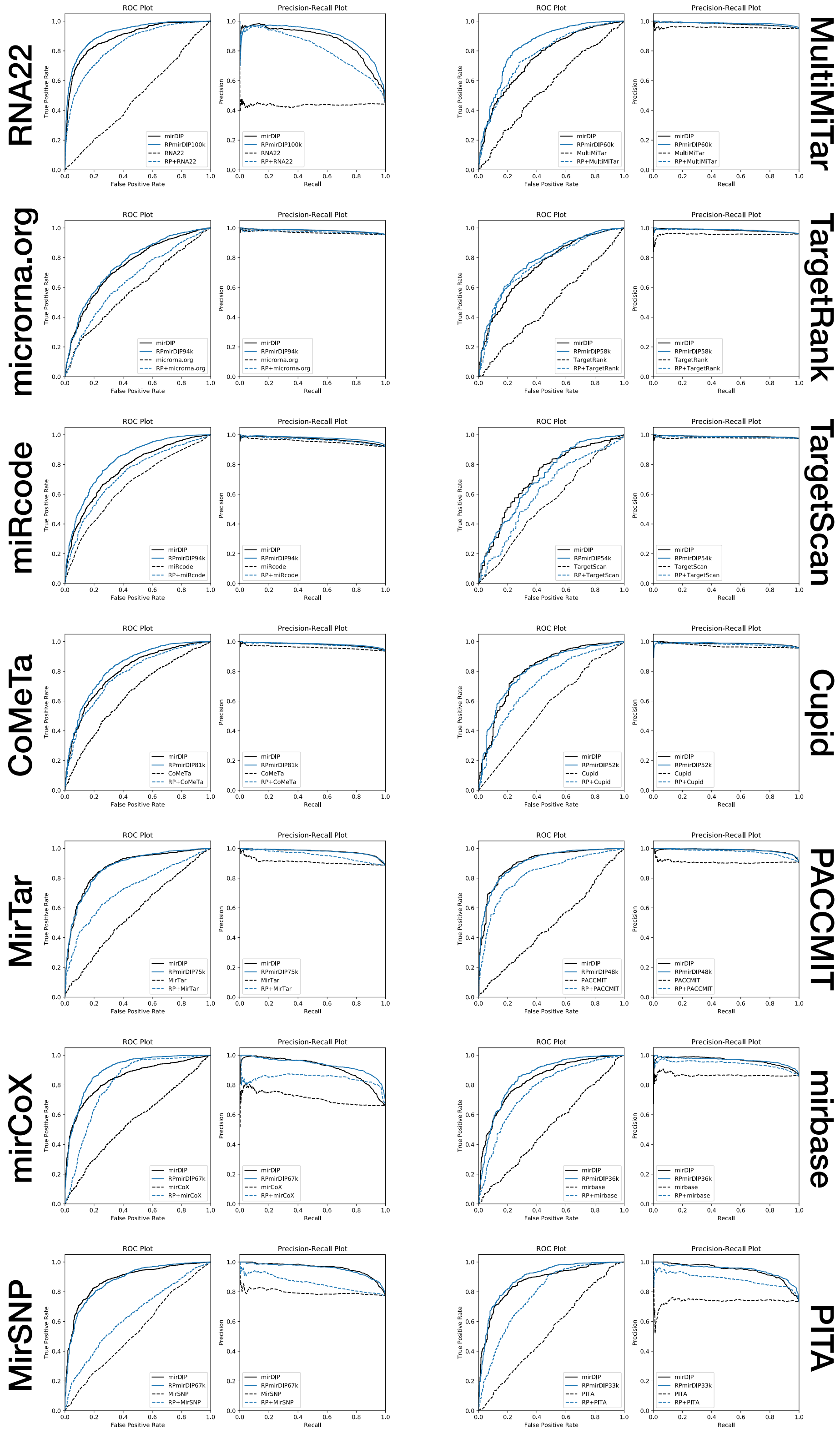

Supplementary Figure 4. ROC and PR curves for the bottom-14 predictors by training set size. To ensure a fair comparison, each of the RPmirDIP predictors compared against each model was only trained on the subset of score pairs available to each method.

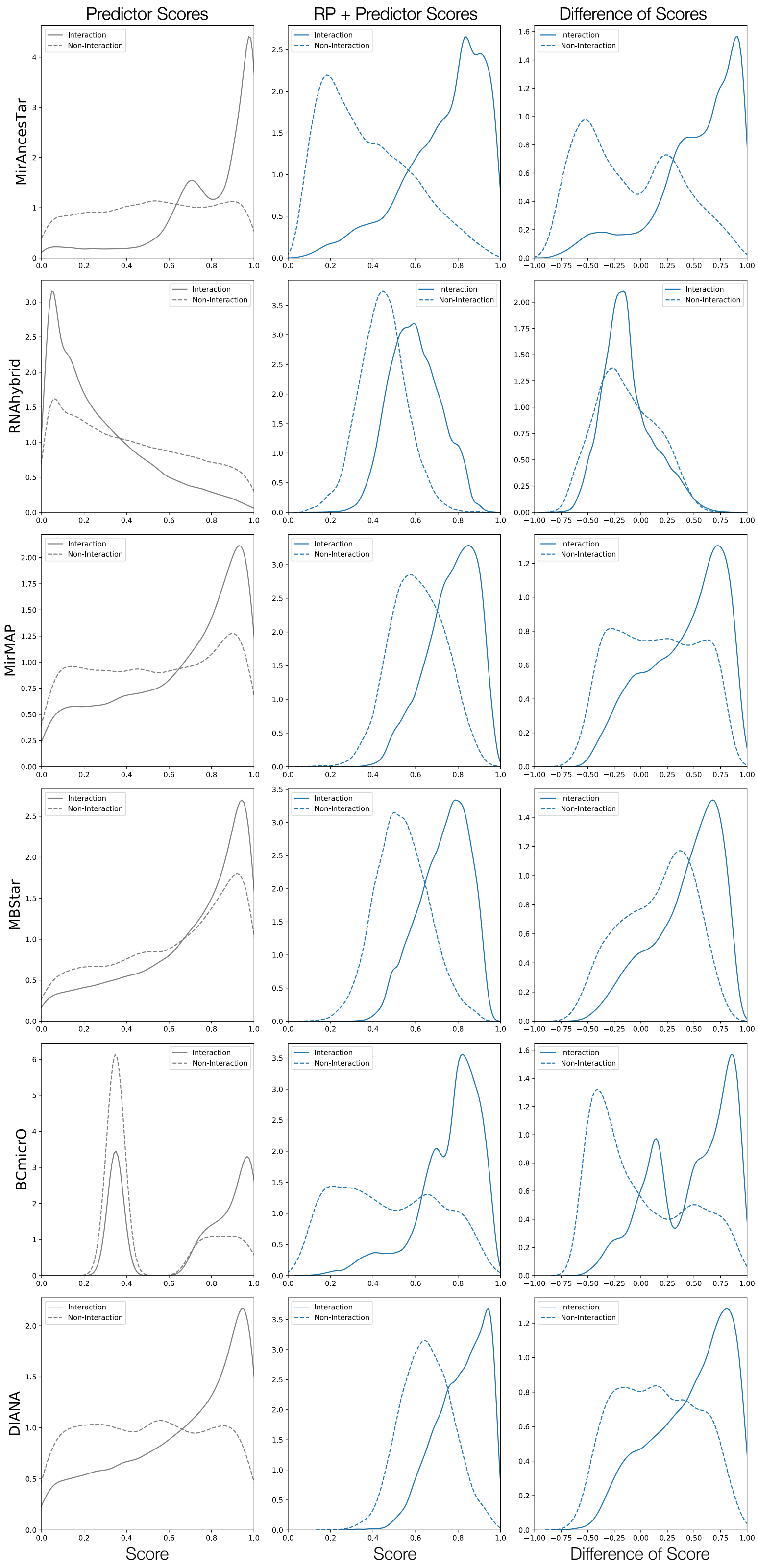

Supplementary Figure 5. Distributions of scores. The left panel plots the distribution of scores of the original predictor, the center panel plots the distribution of scores resultant from the cascaded application of RP, and the right panel plots the difference of scores wherein the predictor's score is subtracted from the RP score.

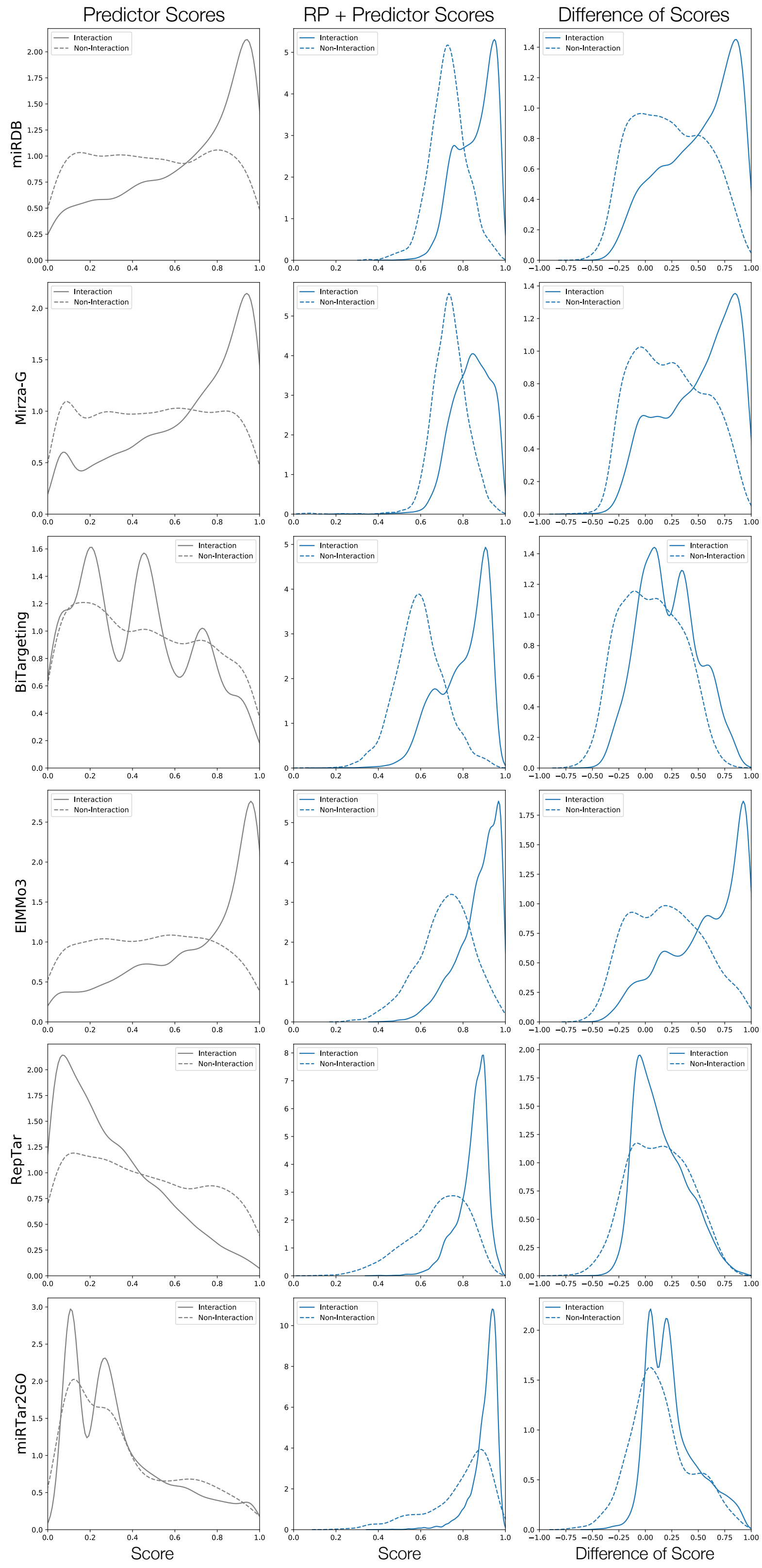

Supplementary Figure 6. Distributions of scores (continued). The left panel plots the distribution of scores of the original predictor, the center panel plots the distribution of scores resultant from the cascaded application of RP, and the right panel plots the difference of scores wherein the predictor's score is subtracted from the RP score.

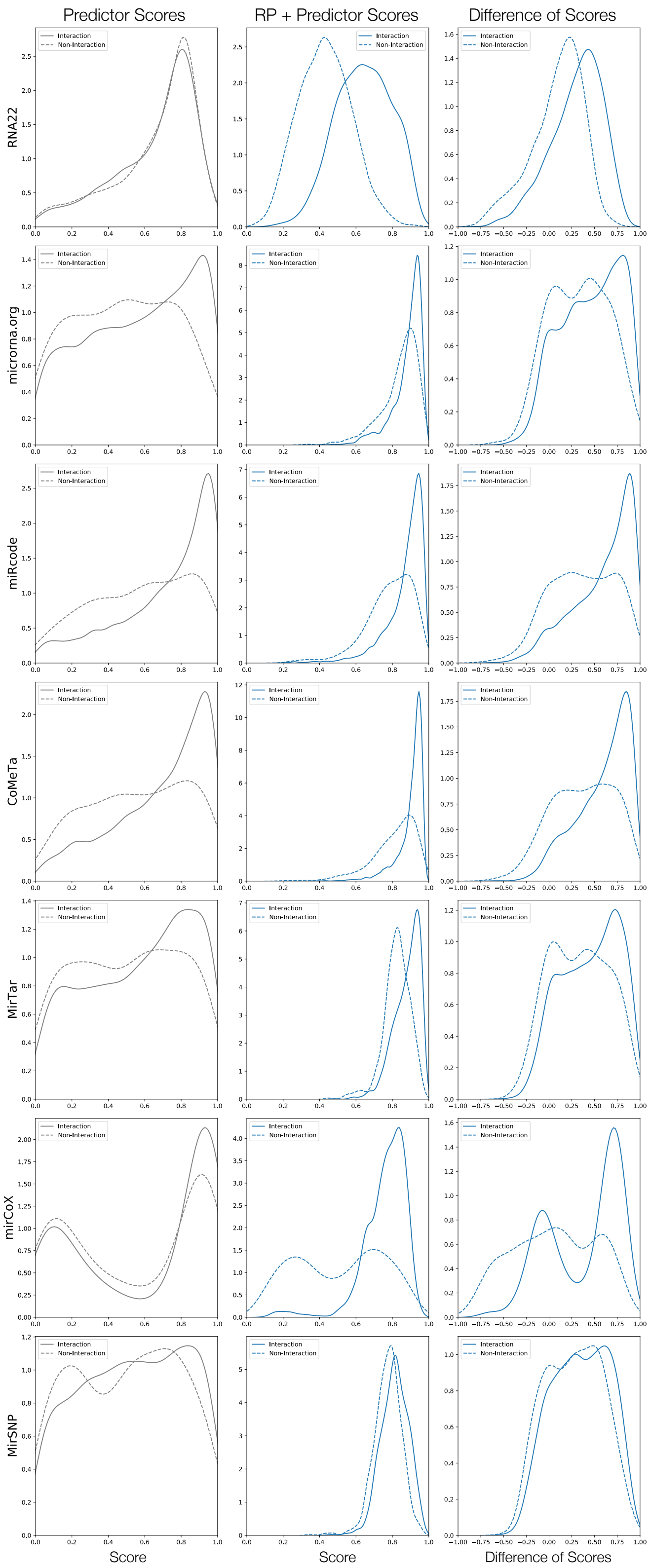

Supplementary Figure 7. Distributions of scores (continued). The left panel plots the distribution of scores of the original predictor, the center panel plots the distribution of scores resultant from the cascaded application of RP, and the right panel plots the difference of scores wherein the predictor's score is subtracted from the RP score.

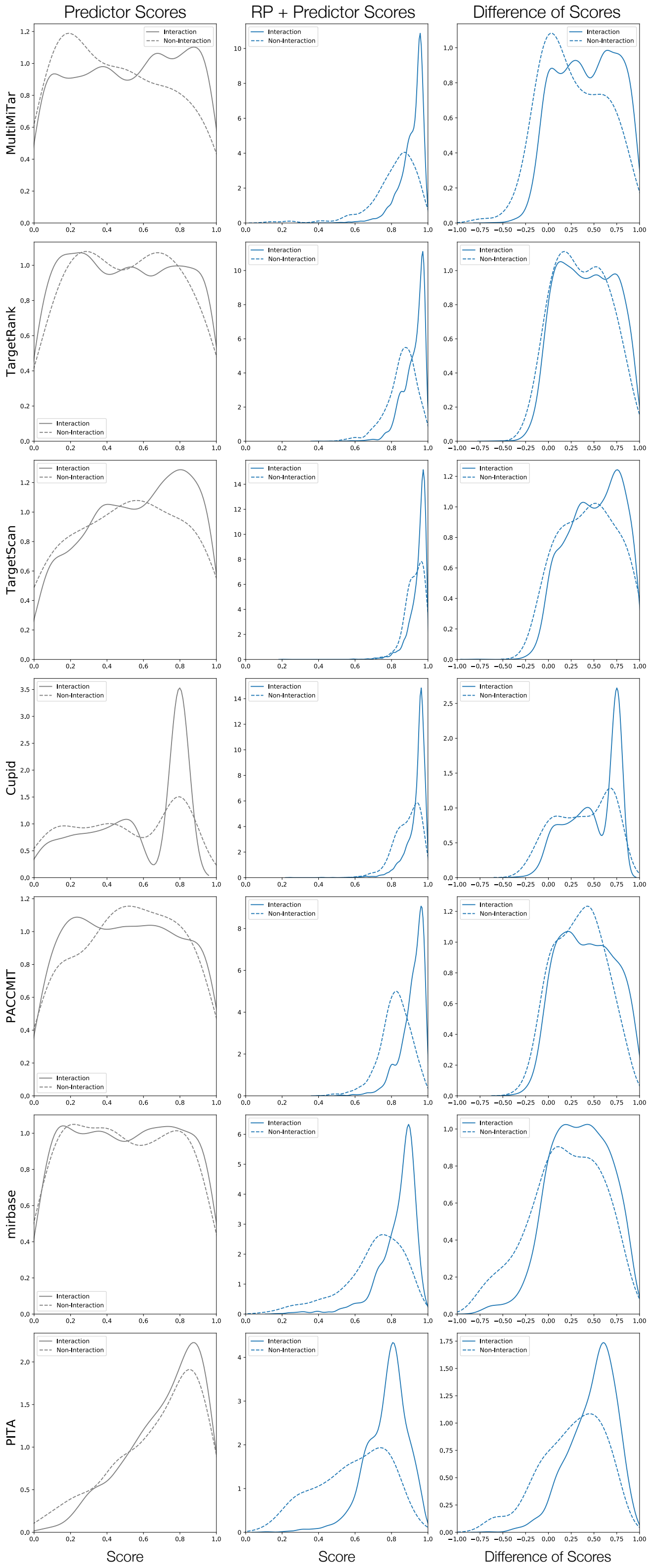

Supplementary Figure 8. Distributions of scores (continued). The left panel plots the distribution of scores of the original predictor, the center panel plots the distribution of scores resultant from the cascaded application of RP, and the right panel plots the difference of scores wherein the predictor's score is subtracted from the RP score.

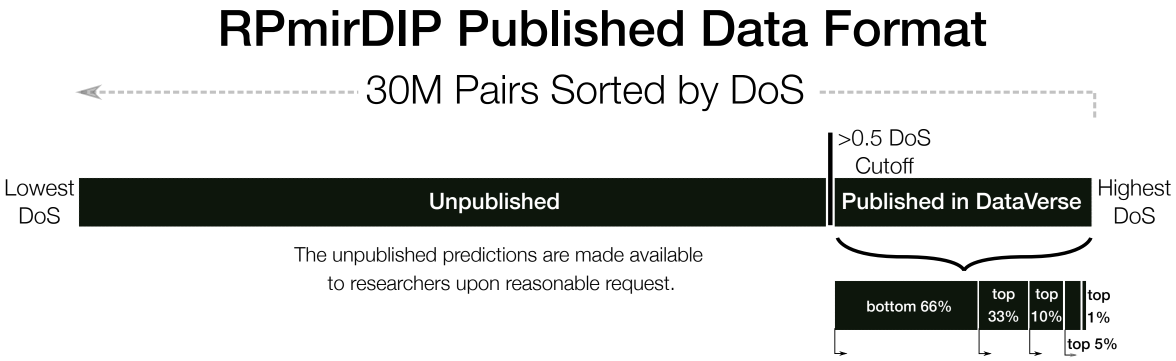

Supplementary Figure 9. Overview of the Published RPmirDIP File Format in the DataVerse. All ∼30 million pairs from the mirDIP database were predicted and sorted in decreasing order based on the DoS. Those pairs with a DoS greater than 0.5 were selected and separated into individual percentile files, one for each of the top-1%, top-5%, top-10%, top-33%, and the remaining bottom-66%. For convenience, a sixth file was created which simply contains all interactions. There exist six files for the two models (RPmirDIP & RPmirDIP*) as well as for the two sorting methods (by DoS & by RPmirDIP(*) score) for a total of 24 files. These files were deposited in a public repository, available at doi.org/10.5683/SP2/LD8JKJ and online at cu-bic.ca/RPmirDIP.
